# Illinois State Medical Society

**Published:** 1858-06

**Authors:** 


### PROCEEDINGS OF MEDICAL SOCIETIES.

### PROCEEDINGS OF THE ILLINOIS STATE MEDICAL SOCIETY.

The Illinois State Medical Society convened in Warner's Hall, city of Rockford, June 1st, 1858, at 10 o'clock. About fifty delegates were present during the meeting. The nominating committee reported as officers for the ensuing year—Dr. H. A. Johnson, of Chicago, *President;* Dr. J. K. Bailey, of Joliet, 1*st Vice President;* Dr. Wm. Lyman, of Rockford, 2*d Vice President;* Dr. J. W. Freer, of Chicago, *Treasurer;* Dr. N. S. Davis, of Chicago, *Permanent Secretary;* Dr. S. McBride, of Decatur, *Recording Secretary.* Neither of the secretaries being present, Drs. Blount and Foote were elected secretaries *pro tem.*

Afternoon, 2 o'clock. A paper was read by Dr. Foote, from Dr. G. W. Phillips, on "purpura hemorrhagica," containing the report of several interesting cases. A resolution was passed, that a committee be appointed to take the necessary steps for incorporating the society. We failed to get the names of this committee. A resolution was also passed, that a committee be appointed to mature a plan and memorialize the Legislature in favor of a law to legalize dissection. Dr. C. N. Andrews, of Rockford, is chairman of that committee.

*June 2d.* After reading the minutes, the next thing in order was the valedictory address of the president, Dr. Goodbrake. The subject of that excellent production was "the causes which retard the improvement of our profession in this State." The 1st conclusion was that of want of preliminary education; 2d, sending medical students to the eastern cities to attend lectures. (The speaker admitted that many years ago it was an evil tolerable only because unavoidable, but he thinks now they may be much better educated in the West, because we have just as able teachers, while we have ample hospitals filled with patients affected with diseases of our own climate, as well as every other almost in the civilized world); 3d, want of discriminative powers, from which arises that false experience which so often misleads the profession; 4th, too little attention to medical jurisprudence both by teachers and students. (He alluded to the many ludicrous blunders often committed by medical men of superior abilities from this negligence); 5th, apathy in encouraging and maintaining county and district medical societies. (He considers them great social stimuli which actuate us to greater exertion for higher attainments, promoting more genial fellowship and unity of effort). The address winds up by an exhortation to the members of the profession not to admit students to their offices who do not possess certain prerequisites in the way of education; to encourage our own medical schools; study medical jurisprudence more thoroughly, so that we may do credit to our profession in this department of it.

Dr. F. K. Bailey, of Joliet, as chairman of committee on practice of medicine, read an erudite and highly-practical report on the subject of disease in Illinois. Quite an interesting debate ensued, in which several members participated.

2 o'clock P. M. The society by invitation visited the public school in West Rockford. It affords us much pleasure to assure the people of Rockford that the whole society were delighted at the prospects so apparent from this well regulated institution.

After reassembling at the hall, a resolution was adopted that enables the society at each session to appoint the time for their next meeting.

A voluntary paper was read by Dr. J. W. Freer, on "a new mode of dressing fractured clavicle." After enumerating the imperfections of all former treatment, he proceeded to explain his plan. The objects being to keep the shoulder up and back, he applies rollers of adhesive strap in the following manner: a pad is first to be placed under the arm, and the right position given to the member and fragments, when a strap of adhesive plaster, two and a half inches wide and a yard and a half long, is attached at its middle to the outside of the middle of the forearm on the affected side, and one end carried up before and the other behind, until they cross over the sound shoulder. Another is to encircle the body and pass over the middle of the affected arm; the hand is then put in a sling, secured by adhesive straps. The dressing seldom needs readjustment until the fracture has entirely united, when they may be laid aside.

A resolution was passed condemnatory of the practice of hiring by the year, month, or week, to attend private families or individuals. It was understood that the resolution did not apply to the attendance for salaries upon public institutions.

At 8 o'clock P. M., the society proceeded to the Metropolitan Hall in East Rockford, where Dr. H. A. Johnson delivered an excellent and acceptable address to a large and intelligent audience.

*June 3d*, 9 o'clock A. M. Society met and proceeded to business. Resolutions were passed recommending the appointment of a committee of three, of which Dr. Stormont, of Grand View, was made chairman, to memorialize the Legislature in favor of a law for the registration of marriages, births and deaths. Dr. Prince, of Jacksonville, was made chairman of a committee to to memorialize the Legislature in favor of a law for the cure and education of the idiotic and insane.

Dr. Chambers, chairman of committee on surgery, read an interesting report, containing the biography of many of our Western surgeons, together with some valuable cases of surgery. A paper was read by Dr. Bailey on "solid concretions in the bowels," with details of a singular case and exhibition of a specimen of this class of accumulations. The thanks of the society were voted to Dr. Johnson for his able address, and a copy requested for publication. Dr. Andrews, of Rockford, read the report of a case of ruptured uterus, in which the rent was so extensive as to permit the escape of a fœtus with the membrane entire, containing a large quantity of liquor amnii, into the peritoneal cavity. The rupture took place without uterine contraction, and probably was the effect of disease in the uterine parietes. The fissure extended from the cervix to the middle of the fundus. Dr. Young, of Aurora, also presented a case of ruptured uterus, occurring during labor, while the head was pressing upon the perinæum. The child was small and dead; it was extracted by forceps, (at least, this was our recollection of the report). The patient recovered and again became pregnant, and at about the same stage of labor, rupture was again produced by the powerful uterine contractions, and what is very remarkable, the patient expired, as Dr. Young expresses it, instantly after the accident. Post-mortem examination revealed an extensive rupture, through which the child and placenta were expelled into the peritoneal cavity.

The committee on nominations reported the following standing and special committees:

*Practical Medicine*—Drs. W. Lyman, chairman, Goodbrake and O. J. Herrick.

*Surgery*—Drs. Powel, chairman, Stearns and Hice.

*Obstetrics*—Drs. Byford, Noble and Young.

*Special Committee on Typhoid Fever* — Dr. Chambers, Charleston.

*On Diseases and Surgery of the Eye*—Dr. Goodwin, Rockford.

*On Anchylosis with Forcible Rupture*—Dr. Freer.

*Committee of Arrangements*—Drs. Trowbridge, King, Chenoweth, Curtis and Moore.

The following gentlemen were elected delegates to the American Medical Association:

Drs. Noble, Harris, Freer, Steele, Nance, McArthur, Edmiston, Bailey, W. Lyman, Luce, L. Clark, Hollister, Young, Chambers and Martin.

A committee was appointed to memorialize the Legislature in favor of a law to legalize dissection—Dr. Andrews, chairman.

Dr. C. W. Clark was appointed to report on "the effect of tobacco on the system."

There was also a committee appointed to report on Drugs and Medicines, but we have forgotten the names of the gentlemen appointed. At 2 o'clock P. M., the society visited the Public School of East Rockford, with which all expressed themselves highly pleased. Various complimentary resolutions were passed, but having no professional interest they are omitted.

The above sketch of the proceedings of the Illinois State Medical Society is intended to embrace most of the important facts without being complete. Doubtless, some things are omitted that would have been interesting, and we would refer the reader to the minutes, as they will be published in the forthcoming volume of transactions.

The society adjourned at 6 o'clock P. M., after accepting the invitation of the ladies of the Female Seminary to attend a festival at that institution, at 8 o'clock P. M. The evening was very stormy, and prevented many from attending who desired to do so. The occasion was one that will not be soon forgotten by the members of the society. Miss Sill, the principal of that new but useful and creditable academy, knows quite as well how to be entertaining to guests as instructive to pupils. She is well sustained by a learned and efficient corps of lady teachers; and oh! what shall we say of the two hundred fair students who sit daily at her feet for instruction? We know of no better way of expressing ourselves than to use the language of ex-president Dr. Goodbrake on quite as perplexing an occasion—"There is no use trying, we cannot do justice to the subject."

Rockford is one of the most lovely little cities we ever visited; and the season of the year, the hospitality of the citizens, the whole-souled liberality of the doctors, and genial feeling prevailing among the members of the society,—all combined to conduce to the utmost the right kind of enjoyment. The only thing that occurred to mar the happiness experienced by all, was the disastrous devastation of the flood on Thursday, but this was more than compensated for by the agreeable detention in such pleasant company.

